# A Brief Overview of Two Major Strategies in Diversity-Oriented Synthesis: Build/Couple/Pair and Ring-Distortion

**DOI:** 10.3389/fchem.2018.00507

**Published:** 2018-10-22

**Authors:** Sihyeong Yi, Begur Vasanthkumar Varun, Yoona Choi, Seung Bum Park

**Affiliations:** Department of Chemistry, CRI Center for Chemical Proteomics, Seoul National University, Seoul, South Korea

**Keywords:** diversity-oriented synthesis, build/couple/pair, ring-distortion, natural product, macrocycle, privileged structure

## Abstract

In the interdisciplinary research field of chemical biology and drug discovery, diversity-oriented synthesis (DOS) has become indispensable in the construction of novel small-molecule libraries rich in skeletal and stereochemical diversity. DOS aims to populate the unexplored chemical space with new potential bioactive molecules via forward synthetic analysis. Since the introduction of this concept by Schreiber, DOS has evolved along with many significant breakthroughs. It is therefore important to understand the key DOS strategies to build molecular diversity with maximized biological relevancy. Due to the length limitations of this mini review, we briefly discuss the recent DOS plans using build/couple/pair (B/C/P) and ring-distortion strategies for the synthesis of major biologically relevant target molecules like natural products and their related compounds, macrocycles, and privileged structures.

## Introduction

Small molecules play an indispensable role in the fields of drug discovery and chemical biology due to their unique features compared to biologics, polymers, and nanoparticles (Samanen, 2013). However, while the knowledge of biological systems has grown in the post-genomic era, the discovery of novel small molecular therapeutics or bioprobes has become more complicated. This can be attributed to advances in chemical biology and drug discovery disclosing novel targets beyond conventional druggable proteins, such as DNA (Hurley, 2002), RNA (Lieberman, 2018; Warner et al., 2018), protein–protein interactions (PPIs) (Scott et al., 2016), and protein–RNA interactions (PRIs) (Hentze et al., 2018), among others. Furthermore, a lack of information regarding the structures and modes of action of these novel targets renders rational drug discovery challenging.

The development of high-throughput screening (HTS) and high-content screening (HCS) enabled rapid and efficient investigation of biological activities to yield existing drug-like compound libraries constructed by combinatorial synthesis (Schreiber, 2000; Tan, 2005; Basso, 2012). However, contrary to expectations, extensive screening exercises against huge compound libraries delivered a relatively small number of new chemical entities, particularly in the case of bioassays for novel undruggable targets or unbiased phenotypic screenings, where rational ligand design is challenging (Burke and Schreiber, 2004; Galloway et al., 2010; Garcia-Castro et al., 2016). This may be due to the limited diversity of conventional drug-like compound libraries, especially in terms of skeletal and stereochemical diversity. Indeed, it should be noted that skeletal diversity is essential for specific binding events with diverse biopolymers bearing three-dimensional (3D) unique binding sites and structural diversity (Kim et al., 2014). In fact, it is not the size of a chemical library that is most important, but the skeletal and stereochemical diversity of its core structures. Thus, there is a huge demand for high-quality compound collections through the efficient construction of drug-like small molecule libraries enriched with molecular diversity, and particularly, skeletal and stereochemical diversity (Spring, 2003).

To meet such demands in molecular diversity, Schreiber et al. introduced the concept of diversity-oriented synthesis (DOS) (Schreiber, 2000). The aim of DOS as a synthetic strategy is to occupy the unexplored parts of chemical space via the efficient synthesis of unique compound collections bearing diversity and complexity in their scaffolds. DOS involves “forward synthetic analysis,” where the products of each step become the branching substrates for subsequent steps (Tan, 2005). Hence, the DOS approach leads to an exponential increase in the molecular diversity of chemical libraries through multiple systematic branching sequences. Indeed, the DOS strategy has attested its capacity and value through the development of various novel therapeutic agents and biological modulators and through advancing biological understandings (Kuruvilla et al., 2002; Kuo et al., 2015; Schreiber et al., 2015; Hideshima et al., 2016; Kato et al., 2016; Plouffe et al., 2016; Wellington et al., 2017; Gerry and Schreiber, 2018). For example, Schreiber *et al*. discovered a novel multistage antimalarial inhibitor, BRD7929, through the extensive screening of their compound library constructed by DOS strategy (Kato et al., 2016), while Park et al. reported a novel leucyl-tRNA synthetase/RagD PPI inhibitor discovered from DOS library (Kim et al., 2016).

**Figure 1:**
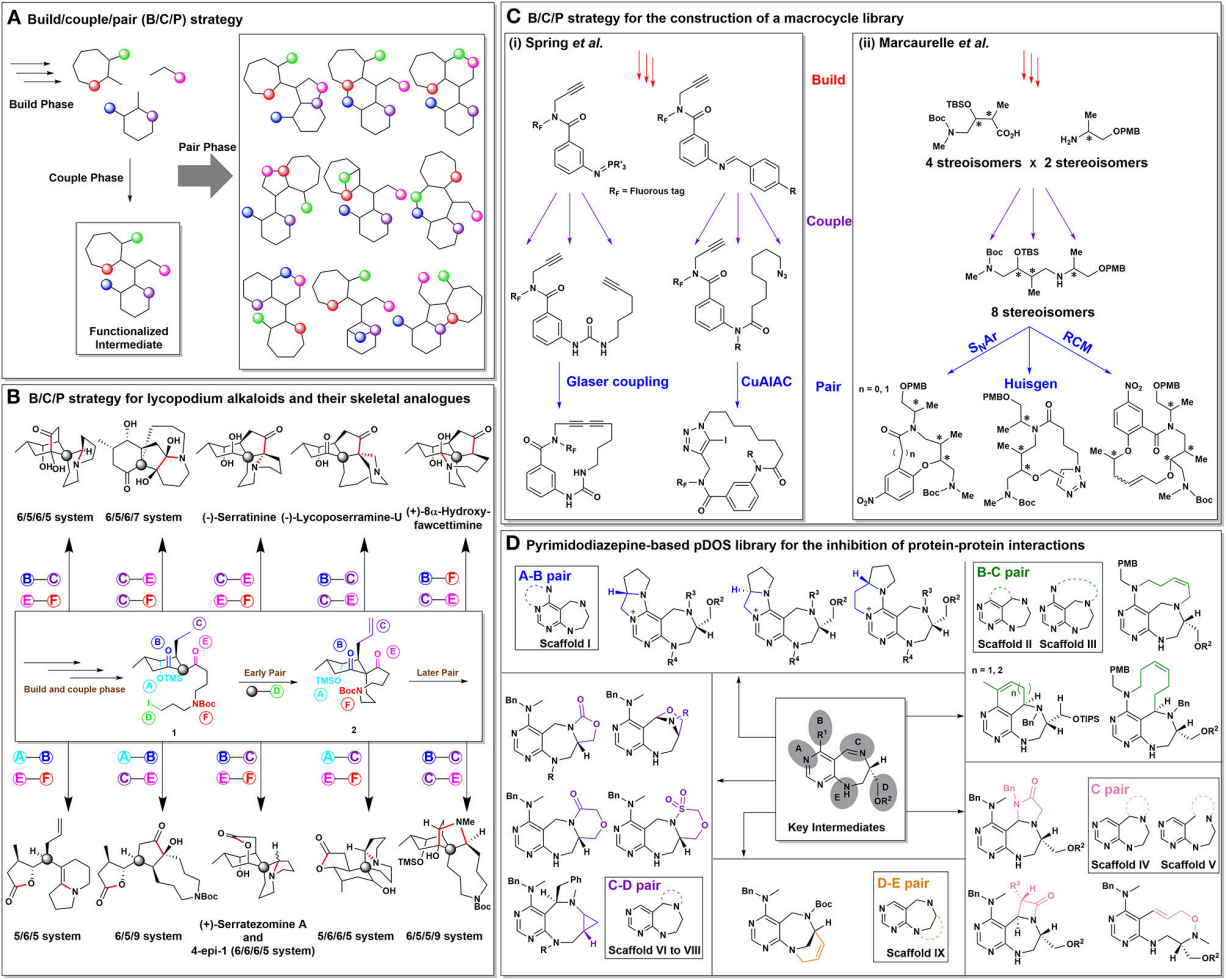
**(A)** A schematic representation of the B/C/P strategy. **(B)** Outline of lycopodium alkaloids and their unnatural scaffolds with their respective pairing patterns. **(C)** Outline of the B/C/P strategy for the construction of libraries consisting of macrocycles and medium-sized rings. **(D)** A pDOS library established via the B/C/P strategy for the inhibition of protein–protein interactions.

Since the DOS concept was introduced, many synthetic pathways have been developed by various research groups to construct efficient chemical libraries (Nielsen and Schreiber, 2008). Among them, the build/couple/pair (B/C/P) strategy is the most widely followed and commonly applicable synthetic strategy. This strategy involves 3 synthetic phases, namely a build phase, a couple phase, and a pair phase (Figure 1A; Burke and Schreiber, 2004). More specifically, the build phase involves the synthesis of a single or multiple key building blocks embedded with suitable functional groups for later-stage coupling reactions. In the couple phase, a variety of intermolecular coupling reactions can be employed to generate a dense array of reactive sites and functional groups on the key building blocks installed during the build phase. Finally, in the pair phase, the intermediates constructed through the build and couple phases take part in intramolecular pairing reactions to yield an array of final products with the desired skeletal and stereochemical diversity (Nielsen and Schreiber, 2008; Kim et al., 2016).

**Figure 2:**
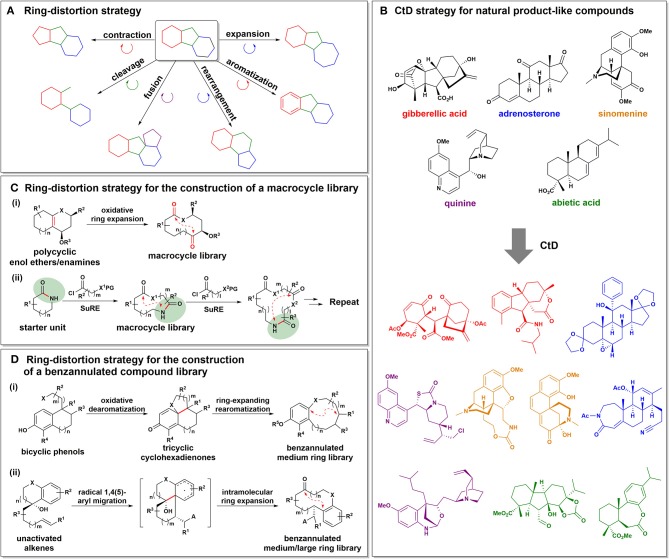
**(A)** Schematic representation of the ring-distortion strategy. **(B)** The complexity-to-diversity (CtD) strategy for the construction of diverse and complex compounds starting from readily available natural products. **(C)** The ring-distortion strategy for the construction of macrocyclic lactone and lactam libraries. **(D)** The ring-distortion strategy for the construction of biologically relevant benzannulated small molecules.

Recently, the ring-distortion strategy has also been developed as a distinctive DOS strategy for the systematic construction of novel small-molecule collections with high structural diversity and complexity. In contrast to the B/C/P strategy, the ring-distortion strategy is distinct in that it pursues molecular diversity via distortion of the existing ring systems through ring-cleavage, ring-expansion, ring-contraction, ring-fusion, ring-rearrangement, ring-aromatization, and combinations of the above (Figure 2A; Huigens et al., 2013).

However, in any DOS strategy, the common structural features of existing bioactive molecules have been widely investigated to grant sufficient biological relevancy to the resulting compounds (Kim et al., 2014). As such, natural products are commonly investigated, and their structural features are considered to be potent sources of information in drug discovery. In addition, macrocycles have received a significant amount of attention in the field of drug discovery due to distinguishable structural features compared to other small molecules. Furthermore, privileged structures, which are common structural motifs in a vast number of bioactive natural products and therapeutic agents, contain novel structural features that secure a high biological relevancy (Evans et al., 1988).

Thus, in this mini review, we present recent advancements in the B/C/P and ring-distortion DOS approaches in the context of natural products, natural product-like compounds, macrocycles, and privileged structures.

## The Build/Couple/Pair (B/C/P) strategy

### Synthesis of natural products and natural product-like compounds via the B/C/P strategy

Natural products play a pivotal role in the search of novel therapeutics. Bioactive natural products tend to have complex 3D polycyclic structures rich in sp^3^ carbons and stereogenic centers, and their inherent bioactivities may provide clues for the design of novel core skeletons with high biological relevancy (Wipf, 2012; Shimokawa, 2014; Chen et al., 2015). Therefore, the efficient construction of natural product libraries and their unnatural analogs can be considered an important DOS strategy.

A team led by Lei proposed that complex molecules such as bioactive natural products can be synthesized via the B/C/P strategy through the pairing of various functional groups present in their structures (Zhang et al., 2014). Selecting lycopodium alkaloids as a model system, they reported the total syntheses of four lycopodium alkaloids and six related unnatural scaffolds. In contrast to other total synthetic approaches, the reported DOS approach allowed the parallel synthesis of unnatural scaffolds, thereby increasing the population of “lycopodium-like” natural products in the unexplored chemical space (Harayama et al., 1974; Ma and Gang, 2004; Chandra et al., 2009). As shown in Figure 1B, chiral intermediate **1** was formed through the build and couple phases, while intermediate **2** was prepared by means of the early pairing phase, which was crucial to the overall synthetic protocol. By encompassing double pairing processes in a sequential manner, the “later pairing phase” allowed the efficient construction of unique core skeletons. To illustrate the power of this B/C/P strategy, stepwise double pairing procedures, such as B–C pairing and E–F pairing in Figure 1B led to the total synthesis of (+)-serratezomine A (6/6/6/5 system) and an unnatural skeletal analog (6/5/6/5 system) of (–)-serratinine. In addition, the double pairing pattern involving A–B pairing followed by C–E or E–F pairing led to the formation of tricyclic compounds (6/5/9 or 5/6/5 systems, respectively). Other pairing patterns leading to the total syntheses of (–)-serratinine, (+)-8α-hydroxyfawcettimine, (–)-lycoposerramine-U, and three tetracyclic unnatural scaffolds were also examined. Overall, this work demonstrated a unique and efficient route to the synthesis of complex natural product-like molecules using the B/C/P strategy.

### Synthesis of macrocycles via the B/C/P strategy

Although various macrocycle-based natural products are known to exhibit therapeutic potential, as a sole structural unit, macrocycles have not been traditionally considered as suitable small molecules for drug discovery screening processes (Schreiber, 2000). However, recent reports have claimed that macrocyclic structures can pre-organize their conformations, which allows improved interactions with extended protein surfaces and subsequent high biological activities (Driggers et al., 2008; Villar et al., 2014). As such, numerous DOS strategies have been pursued to construct structurally and functionally diverse macrocycles (Madsen and Clausen, 2011; Collins et al., 2016).

For the efficient construction of libraries containing a diverse array of macrocycles, Spring et al. developed advanced B/C/P approaches (Beckmann et al., 2013; Nie et al., 2016). These B/C/P approaches not only allowed diversification in the multi-dimensional pattern, but also resulted in the judicious modification of the chemical structures following the pairing phase (Figure 1Ci). Using an advanced B/C/P approach, they also reported the synthesis of a library containing 73 macrocycles having 59 different scaffolds (Beckmann et al., 2013). In this case, the build phase involved the synthesis of fluorous-tagged azido compounds, which were converted *in situ* into the corresponding pluripotent aza-ylides. These aza-ylides were then coupled with suitable appendages to facilitate the subsequent pairing reactions. Similarly, in 2016, they reported the synthesis of 45 diverse macrocyclic compounds of various sizes, ranging from 15- to 33-membered rings (Nie et al., 2016). In this case, the imine moieties branching out from the aza-ylides served as second-line building blocks for diversification of the macrocycle library. The introduction and subsequent modification of the fluorous tag and other reactive sites in these macrocycles could therefore improve the efficiency as well as skeletal diversity of the library synthesis.

Moreover, Marcaurelle et al. utilized an aldol-based B/C/P strategy to construct a library containing in excess of 30,000 compounds, which were based on a variety of skeletons ranging from 8- to 14-membered rings, of which 14,400 compounds were macrolactams aimed at the discovery of novel histone deacetylase inhibitors (Figure 1Cii; Marcaurelle et al., 2010). Notably, this study presented an excellent example of the DOS strategy to demonstrate its power and efficiency for the highly systematic construction of small-molecule libraries with maximized architectural complexity.

### The B/C/P strategy in the pdos pathway

A clear definition of privileged structures was made in a seminal article on drug discovery methods reported by Evans et al. (1988). More specifically, they stated that “privileged structures are capable of providing useful ligands for more than one receptor and that judicious modification of such structures could be a viable alternative in the search for new receptor agonists and antagonists.” Based on the concept of modification around privileged structures, a number of groups have reported various bio-relevant compounds, with many successfully delivering clinical candidates as well as FDA-approved drugs (Mason et al., 1999; Nicolaou et al., 2000a,b,c; Brohm et al., 2002; Kissau et al., 2003; Newman, 2008). For example, Nicolaou et al. published a series of articles on the combinatorial library syntheses of natural product-like compounds in which the benzopyran skeleton was employed as a privileged structure (Nicolaou et al., 2000a,b,c). In this context, the construction of a DOS library derived from privileged structures can be considered crucial to accessing highly biologically relevant molecular diversity (Kim et al., 2014).

We envisioned that incorporating these privileged structures into polyheterocycles enhances the biological relevancy of the resulting compounds with pre-defined conformations, which may be beneficial for specific binding with biopolymers due to the prepaid entropic penalty (Oh and Park, 2011; Kim et al., 2014; Lenci et al., 2016). Hence, within the theme of DOS, our group introduced a novel design strategy, namely “privileged substructure-based diversity-oriented synthesis” (pDOS), which aims to populate the chemical space with privileged substructure-embedded polyheterocycles (An et al., 2008; Oh and Park, 2011; Zhu et al., 2012; Kim et al., 2013, 2014). In particular, the systematic construction of diverse sp^3^-rich 3D polyheterocycles containing privileged substructures has been emphasized since their rigid and diverse frameworks can selectively bind with biopolymers to induce conformational changes and subsequent functional modulation. Thus, a small-molecule library constructed by the pDOS strategy could be considered an excellent resource for the discovery of specific modulators of protein–protein and protein–DNA/RNA interactions.

In addition, we recently reported a pDOS library in which pyrimidodiazepines were employed as the privileged substructure (Kim et al., 2016). We found that the 6/7-bicyclic pyrimidodiazepine system demonstrated a significantly higher conformational flexibility with more reactive sites compared to those of pyrimidine-embedded 6/6 or 6/5 systems. In this case, the build and couple phases produced key pyrimidodiazepine-based intermediates containing five orthogonal reactive sites. In the pair phase, each reactive site was paired to produce 16 different polyheterocycles containing the pyrimidodiazepine substructure and with a high degree of 3D skeletal complexity in nine distinct scaffolds. As shown in Figure 1D, A–B pairing and B–C pairing led to the synthesis of tetracyclic and tricyclic compounds, respectively (scaffolds I–III). Due to the dual (i.e., electrophilic and nucleophilic) nature of the imine moiety, the C pairing allowed the synthesis of scaffolds IV and V. Using the C–D and D–E pairing combinations, scaffolds VI–IX were also constructed. Based on our HTS screening endeavors against this pDOS library, we identified aziridine-containing pyrimidodiazepines from scaffold VIII (constructed through C–D pairing) as a novel small-molecule inhibitor of the leucine tRNA synthetase (LRS)–RagD protein–protein interaction.

## The ring-distortion strategy

### Synthesis of natural product-like compounds via the ring-distortion strategy

For the construction of natural product-like compound collections, Hergenrother et al. developed a novel approach starting from natural products, known as the complexity-to-diversity (CtD) strategy (Huigens et al., 2013; Rafferty et al., 2014; Garcia et al., 2016). In this approach, the molecular frameworks of readily available natural products were converted into structurally complex and diverse core skeletons through various chemoselective ring-distortion reactions (Figure 2B). As natural products exhibit an inherent structural complexity with defined stereochemistry (Clardy and Walsh, 2004), the resulting core skeletons derived from natural products tend to be structurally and stereochemically more complex and distinct compared to existing compound collections. In their initial report on the CtD strategy, gibberellic acid, quinine, and adrenosterone were employed as synthetic starting points, and were transformed into 19, 12, and 18 different scaffolds, respectively, through various ring-distortion reactions (3 reaction steps on average; Huigens et al., 2013). The subsequent application of traditional diversification strategies to final scaffolds therefore allowed the construction of a 119-membered highly complex compound library. They also applied the CtD strategy to other readily available natural products such as abietic acid and sinomenine, which afforded 84 and 65 complex compounds, respectively (Rafferty et al., 2014; Garcia et al., 2016). Chemoinformatic analysis of the resulting compound collections obtained using the CtD strategy demonstrated a higher skeletal complexity compared to conventional compound collections in terms of higher fractions of sp^3^-hybridized carbon atoms (F_sp3_), lower clogP values, and greater numbers of stereocenters.

### Synthesis of macrocycles via the ring-distortion strategy

For the systematic construction of diverse macrocycles, several DOS approaches utilizing ring-distortion reactions (and in particular, ring-expansion reactions) have been pursued (Kopp et al., 2012; Kitsiou et al., 2015; Stephens et al., 2017, 2018). For example, Tan et al. reported an efficient oxidative ring-expansion strategy for the construction of diverse macrocyclic small molecule collections (Figure 2Ci; Kopp et al., 2012). Interestingly, easily accessible polycyclic enol ethers or enamines containing bridging double bonds were found to smoothly undergo oxidative cleavage to generate various macrolactones and macrolactams, regardless of substrate effects, such as ring size, substituents, and stereochemistry. Subsequent transformations using functional handles in the macrocyclic scaffolds afforded additional structural diversity. In addition, the chemoinformatic analysis of 32 unprecedented macrocyclic compounds using principal component analysis (PCA) and principal moments of inertia (PMI) analysis illustrated the possibilities of the resulting macrocycles to modulate novel biological targets through occupying unique chemical space distinct from the current synthetic drugs.

Moreover, the successive ring-expansion (SuRE) strategy described by Unsworth et al. led to the generation of structurally diverse macrocyclic lactams and lactones in a sequential manner (Kitsiou et al., 2015; Stephens et al., 2017, 2018). As shown in Figure 2Cii, the amide functionality present in the cyclic starter unit enabled coupling with the linear fragment via an acylation reaction, and subsequent deprotection and ring-opening along with chain incorporation yielded the ring-expanded product. The key strength of the SuRE method is that the same coupling and ring-expansion sequence can be repeated as the reactive amide functionality is regenerated in the product. Using this simple SuRE strategy, a functionalized macrocycle library was successfully constructed.

### Synthesis of biologically relevant benzannulated compounds via the ring-distortion strategy

Benzannulated medium/macro- or bridged rings are common structural moieties in a number of bioactive natural products and pharmacologically significant synthetic compounds such as penicillide, zeranol, and rifampin (Salituro et al., 1993; Fürstner et al., 1999; Yu and Sun, 2013; Hussain et al., 2014). In this context, Tan et al. developed an efficient biomimetic ring-expansion approach to construct diverse benzannulated medium-sized rings via an oxidative dearomatization and ring-expanding rearomatization sequence (Figure 2Di; Bauer et al., 2013). This strategy involves the oxidative dearomatization of bicyclic phenol precursors to provide polycyclic cyclohexadienones and a subsequent ring-expansion driven by rearomatization of the phenol ring to afford benzannulated medium-sized rings. The structural and physicochemical similarities between the resulting 47 scaffolds and benzannulated medium ring-based natural products were confirmed by PCA analysis.

Furthermore, Liu et al. reported a radical-based diversity-oriented synthetic approach for the fabrication of 37 discrete benzannulated medium/macro- or bridged-rings in a stereoselective manner (Figure 2Dii; Li et al., 2016). In this strategy, the radical 1,4- or 1,5-aryl migration of unactivated alkenes and subsequent intramolecular ring-expansion provided benzannulated medium or large rings. Additional ring-distortion reactions of the resulting core skeletons afforded novel medium-sized and medium-bridged rings with high regio- and stereoselectivities. PCA analysis and preliminary biological studies confirmed the significant biological relevance of this compound collection.

## Conclusion

In this mini review, we briefly emphasized the important roles of diversity-oriented synthesis (DOS) in the field of drug discovery and chemical biology, and introduced the most common DOS strategies for the construction of novel small molecule libraries with maximized molecular diversity. We also discussed two key diversity-oriented synthetic approaches (i.e., the build/couple/pair (B/C/P) strategy and the ring-distortion strategy) and visualized how each strategy allows design of the resulting scaffolds with high biological relevancy via the incorporation of key structural elements such as bioactive natural products, macrocycles, and privileged structures. We concluded that both the B/C/P strategy and the ring-distortion strategy are powerful approaches for the creation of a number of diverse and complex scaffolds in an efficient manner. The combination of DOS-based molecular diversity and unbiased phenotypic screening may shed light on the unraveled signaling pathways and other intricate biological processes by allowing the sustainable supply of new drug candidates and chemical probes.

## Author contributions

SY, BV, and YC contributed equally to this manuscript. SY, BV, and YC collected the related references and prepared the manuscript. SP directed the preparation of this manuscript. All authors critically reviewed the text and figures prior to submission.

### Conflict of interest statement

The authors declare that the research was conducted in the absence of any commercial or financial relationships that could be construed as a potential conflict of interest.
